# Is the segmental wall motion predictor of quality of life changes two years after coronary artery by-pass surgery?

**DOI:** 10.1186/1749-8090-8-S1-P81

**Published:** 2013-09-11

**Authors:** V Peric, S Sovtic, A Jovanovic, R Stolic, S Lazic, Z Marcetic, D Djikic, P Otasevic

**Affiliations:** 1 University of Pristina, School of Medicine, Internal Clinic, Kosovska Mitrovica, Serbia; 2 Dedinje Cardiovascular Institute, Belgrade, Serbia

## Background

The advantage in predictive value of coronary artery bypass surgery (CABG), as the therapeutic treatment in patients with coronary artery disease with low left ventricle ejection fraction (EF) is well known. Influence of abnormal segmental wall motion on the reduction of EF is well known. However, it is not quite clear, what is the relation between abnormal segmental wall motion and quality of life (QOL) after CABG.

## Methods

We administered the Nottingham Health Profile Questionnaire part I in a consecutive series of patients (243 pts, 195 men) who underwent elective CABG. The questionnaire was distributed before and two years after CABG, to all patients. EF was determined by echocardiography, after Simpson method. Segmental wall motion was determined by echocardiography and ventriculography.

## Results

Preoperatively, abnormal segmental wall motion was found in 188 (77%) patients, and normal at 55 (23%) patients. Patients with abnormal segmental wall motion had significantly the lower EF (52.89 ± 8.11 vs. 40.94 ± 10.93, p<0.001). After CABG, QOL was significantly improved in both examined groups (p<0,01). There was no significant correlation between the amount of QOL changes two years after CABG and segmental wall motion (p>0,05). Univariate and multivariate logistic regression showed that abnormal segmental wall motion was not the independent predictor of the patients improved or worsened by operation.

## Conclusions

Two years after CABG, QOL was significantly improved and that improvement was not related to segmental wall motion. Abnormal segmental wall motion was not the predictor of QOL changes two years after CABG.

